# Trichostatin A specifically improves the aberrant expression of transcription factor genes in embryos produced by somatic cell nuclear transfer

**DOI:** 10.1038/srep10127

**Published:** 2015-05-14

**Authors:** Kimiko Inoue, Mami Oikawa, Satoshi Kamimura, Narumi Ogonuki, Toshinobu Nakamura, Toru Nakano, Kuniya Abe, Atsuo Ogura

**Affiliations:** 1 Bioresource Center, RIKEN, 3-1-1 Koyadai, Tsukuba, Ibaraki, 305-0074 Japan; 2 Graduate School of Life and Environmental Sciences, University of Tsukuba, 1-1-1 Ten-noudai, Tsukuba, Ibaraki, 305-8572 Japan; 3 Department of Pathology, Medical School and Graduate School of Frontier Biosciences, Osaka University, 2-2 Yamadaoka, Suita, Osaka, 565-0871 Japan

## Abstract

Although mammalian cloning by somatic cell nuclear transfer (SCNT) has been established in various species, the low developmental efficiency has hampered its practical applications. Treatment of SCNT-derived embryos with histone deacetylase (HDAC) inhibitors can improve their development, but the underlying mechanism is still unclear. To address this question, we analysed gene expression profiles of SCNT-derived 2-cell mouse embryos treated with trichostatin A (TSA), a potent HDAC inhibitor that is best used for mouse cloning. Unexpectedly, TSA had no effect on the numbers of aberrantly expressed genes or the overall gene expression pattern in the embryos. However, in-depth investigation by gene ontology and functional analyses revealed that TSA treatment specifically improved the expression of a small subset of genes encoding transcription factors and their regulatory factors, suggesting their positive involvement in *de novo* RNA synthesis. Indeed, introduction of one of such transcription factors, Spi-C, into the embryos at least partially mimicked the TSA-induced improvement in embryonic development by activating gene networks associated with transcriptional regulation. Thus, the effects of TSA treatment on embryonic gene expression did not seem to be stochastic, but more specific than expected, targeting genes that direct development and trigger zygotic genome activation at the 2-cell stage.

Cloning by somatic cell nuclear transfer (SCNT) is a unique reproductive technique for medical, agricultural and basic biology used for investigating reprogramming mechanisms in mammalian preimplantation embryos. However, the developmental ability of SCNT-derived embryos (henceforth SCNT embryos) in general is much lower than that of normally fertilized eggs because of developmental arrest at the pre- and postimplantation stages^1^. This is also the case with mouse cloning, which shows about 50% of SCNT embryos arresting their development before the blastocyst stage, although efficiencies can vary with the experimental conditions^4^. Interestingly, in our previous studies, the rates of 4-cell stage development (per 2-cell stage) showed good correlation with the rates of development to term per embryos transferred^5^. In other words, the outcome of each cloning experiment could be estimated by observing embryos as early as at the 4-cell stage.

The maternal–zygotic transition (MZT) is an essential process for embryo development. Thus, mouse embryos commence major zygotic gene activation (ZGA) at the 2-cell stage and they never develop further without it. Therefore, development of SCNT embryos into the 4-cell stage described above might reflect the efficiency of ZGA in these embryos. Concomitantly with this process, a number of oocyte-specific transcripts are degraded and replaced with transcripts newly synthesized in zygotes^7^. Many zygotic genes play key roles in further embryonic development, and their expression failure may cause developmental arrest at certain stages of development^8^. MZT is a highly organized phenomenon and progresses in a sequential manner that is conserved between species^9^.

Two research groups have shown that treatment of SCNT embryos with the histone deacetylase (HDAC) inhibitor, trichostatin A (TSA), significantly improved birth rates in mice^1^. It has been demonstrated that treatments with HDAC inhibitors were also effective for SCNT cloning success in several other species although the effect varied with species^4^. In mice, SCNT embryos treated with HDAC inhibitors significantly improved the rate of development beyond the 2-cell stage. Upregulation of the expression levels of the key developmental genes in TSA-treated SCNT embryos was considered to be one of the major reasons for the improvements in development. In one case, Li *et al.* reported increased expression levels of pluripotent-related genes and decreased expression levels of DNA methylation-related genes in blastocyst-stage mouse embryos^5^. Immunocytochemical studies revealed that SCNT embryos treated with HDAC inhibitors underwent *de novo* RNA synthesis and activated ribosomal RNA genes more efficiently than did untreated SCNT embryos^8^.

From this background, we expected that some zygotically activated genes (ZAGs) might give a good indication of cloning efficiency. The identification of such genes and information regarding their transcriptional control could also provide valuable clues for understanding genomic reprogramming following SCNT. However, in the previous studies, the analysis of gene expression patterns was limited because quantitative reverse transcription polymerase chain reaction (RT–PCR) or the detection of bromodeoxyuridine labelling had been used. Therefore, systematic gene expression analysis is necessary for further understanding of the effects of HDAC inhibitors on gene expression in SCNT embryos. Here, we used microarray analysis to analyse the global gene expression profiles in mouse 2-cell SCNT embryos. We paid special attention to the effects of TSA treatment, which significantly ameliorates the developmental arrest of SCNT embryos at ZGA (2-cell) and thus improves cloning efficiency by 5- to 10-fold. Interestingly, although no clear-cut changes were found in gene expression profiles as a whole after TSA treatment, a detailed gene ontology (GO) analysis revealed specific improvements in the expression of genes regulating transcription in early embryos.

## Results

### Comparison of global gene expression profiles in unfertilized oocytes, *in vitro* fertilization (IVF)-derived embryos, SCNT embryos with or without TSA treatment and donor cumulus cells

**Figure 1:**
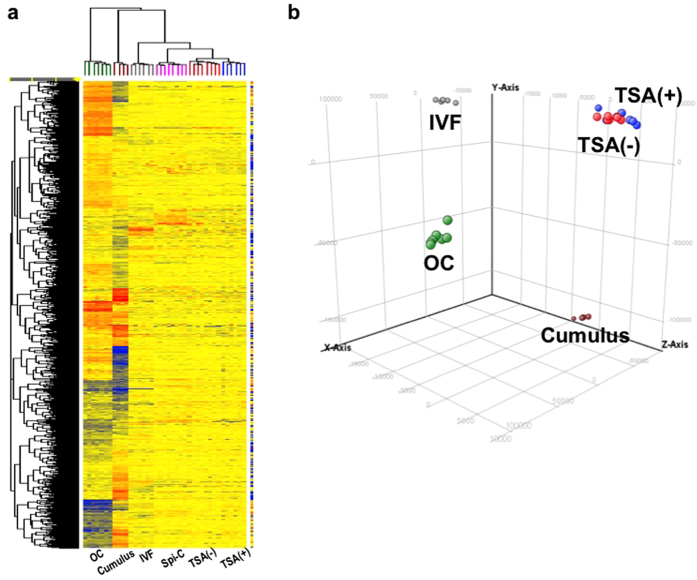
Gene expression profiles of oocytes, 2-cell stage embryos and nuclear donor cells. Hierarchical clustering (**a**) and principal component analysis (PCA) (**b**) of gene expression data in recipient oocytes (OC, green, n = 7), 2-cell stage IVF-derived (IVF, grey; n = 6), nontreated SCNT (TSA(–), red; n = 8), TSA-treated SCNT (TSA(+), blue; n = 6) embryos and cumulus cells (Cumulus, brown, n = 4). SCNT embryos showed gene expression profiles entirely different from those of normally fertilized IVF-derived embryos, irrespective of TSA treatment.

To compare the gene expression profiles of different embryos/cell groups, we employed multivariate analyses: hierarchical clustering and principal component analysis (PCA). We generated cumulus cell-derived SCNT embryos in the absence or presence of TSA for 8 h postactivation (TSA(–) and TSA(+); n = 8 and 6, respectively). Then, their global gene expression patterns at the 2-cell stage were analysed in comparison with those of unfertilized oocytes (OC, n = 7), genetically matched IVF-derived 2-cell embryos (IVF, n = 6) and donor cumulus cells (Cumulus, n = 7) using an Agilent Whole Mouse Genome 44 K array (Agilent Technologies, Palo Alto, CA, USA). Figure 1a shows a hierarchical clustering dendrogram of OC, IVF, TSA(–), TSA(+) and Cumulus samples for 27,954 genes (probes), which were selected by the coefficient of variance of < 50% within each group. All embryo or cell groups were clearly separated into each classification. There was a dramatic change in the gene expression pattern from OC to IVF, as reported in a previous study on the gene expression profiles of mouse preimplantation embryos^9^. These results indicated that our experimental system was accurate enough for minimizing the variation within the group and for detecting alterations in the gene expression profiles between groups. PCA using the same gene set also confirmed the similarity of TSA(+) embryos to TSA(–) embryos and their clear separation from IVF embryos (Fig. 1b). Thus, multivariate analyses of the global gene expression profiles provided no evidence for any particular improvement in gene expression in SCNT embryos following TSA treatment.

### Lack of improvement in SCNT-specific aberrations in gene expression by TSA treatment

**Figure 2:**
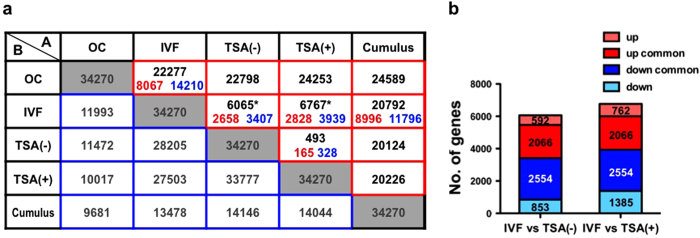
A cross-table of *post hoc* statistical analysis (*P* < 0.01) of OC, IVF, TSA(–), TSA(+) and Cumulus. (**a**) The 34,270 genes shown in the grey boxes were evaluated as significantly different between at least two groups (one-way ANOVA). Numbers in red boxes indicate differentially expressed genes (DEGs) between A and B (Tukey HSD *post hoc* test), and the genes found not to be differentially expressed are indicated in blue boxes. Red and blue numbers within red boxes indicate up- and downregulated genes, respectively (A/B). Numbers marked by asterisks are shown in detail in Fig. 2b. There were no clear differences between TSA(–) and TSA(+) in the numbers of DEGs. (**b**) Breakdown of DEGs between IVF-derived and TSA(–) or TSA(+). Dark- and pale-coloured columns indicate commonly or differentially upregulated or downregulated genes between both groups, respectively. Of these, 1,445 genes (592 upregulated and 853 downregulated) and 2,147 (762 upregulated and 1,385 downregulated) genes were DEGs specific for TSA(–) and TSA(+) SCNT embryos, respectively, and 1,445 DEGs in IVF vs. TSA(–) embryos were expected to be corrected by TSA treatment. Both DEG groups were subjected to GO analysis by DAVID bioinformatics as shown in Supplementary Tables S2 and S3.

We then extracted differentially expressed genes (DEGs) among the OC, IVF, TSA(–), TSA(+) and Cumulus groups to identify any aberrantly expressed genes in SCNT embryos. In this analysis, 39,870 genes that showed more than 10 units in their signal intensities were used. The cross-table from one-way analysis of variance (ANOVA) and *post hoc* statistical analysis (*P* < 0.01) is shown in Fig. 2a. In the direct comparison between TSA(–) and TSA(+), we identified only 493 DEGs (165 upregulated and 328 downregulated genes in TSA(+)). The fact that a very small number of genes changed between these groups is coincident with the result of hierarchical clustering and PCA shown in Fig. 1a,b, which show that the expression pattern of TSA(–) and TSA(+) was similar. We also found that 6,065 and 6,767 genes were expressed differentially between the two groups of IVF vs. TSA(–) and IVF vs. TSA(+), respectively, indicating that TSA treatment had no beneficial effect on the number of DEGs. Approximately 70% of genes marked as DEG in TSA(–) and TSA(+) embryos were common to these two groups (4,620 genes included 2,066 up- and 2,554 downregulated genes in SCNT embryos, respectively) (Fig. 2b). By excluding these common DEGs, we calculated that there were 1,445 (592 upregulated and 853 downregulated) and 2,147 (762 upregulated and 1385 downregulated) genes with alterations in their expression levels according to the absence or presence of TSA, respectively (Fig. 2b). These gene sets were subjected to GO analyses as described below.

Next, we investigated SCNT-specific aberrations in gene expressions from the perspective of the MZT. First, we identified 8,067 ZAGs, which were transcripts with a significantly higher expression in IVF than OC (*P* < 0.01) and 14,210 zygotically degraded genes (ZDGs), which were transcripts with a significantly lower expression in IVF than OC, (*P* < 0.01, Fig. 2a). We examined whether the numbers of ZAGs or ZDGs could explain the improved development of TSA-treated SCNT embryos. As shown in the Venn diagrams in Supplementary Fig. S1, there were no significant differences in the numbers of ZAGs or ZDGs between the TSA(–) and TSA(+) groups (6,555 vs. 6,488 genes for ZAGs and 12,579 vs. 13,076 genes for ZDGs, respectively). We further performed a similar analysis based on the nuclear donor cells. We identified 11,796 donor-repressed genes and 8,996 donor-activated genes by comparing the gene expression patterns of cumulus cells with IVF embryos (Fig. 2a, Supplementary Fig. S1). There were no significant differences in the numbers of donor-repressed genes or donor-activated genes between the TSA(–) and TSA(+) groups (9,879 vs. 9,557 for donor-repressed genes and 7,728 vs. 7,777 for donor-activated genes, respectively). Thus, any analytical methods based on the numbers of DEGs did not account for the improved development of TSA-treated SCNT embryos.

### Gene ontology analysis of upregulated or downregulated genes in SCNT embryos

Next we evaluated the gene functions of DEGs specifically identified for TSA(–) or TSA(+) SCNT embryos. Gene functions were searched using DAVID Bioinformatics Resources 6.7^1^. At first, we examined GO terms associated with the 493 genes of direct comparison between TSA(–) and TSA(+) shown in Fig. 2a, and only one GO term “regulation of T cell proliferation” was identified (*P* < 0.01, Supplementary TableS1). Unexpectedly, this function did not seem to be significant in embryonic development during the early stage, and we searched GO terms for other categories. For TSA(–) and TSA(+) embryos, we identified 6,065 and 6,767 DEGs compared with IVF embryos, respectively, as described above (Fig. 2a). Of them, 1,445 (592 upregulated and 853 downregulated) and 2,147 (762 upregulated and 1,385 downregulated) DEGs were specific for TSA(–) and TSA(+) embryos, respectively (Fig. 2b). The DEGs of each category were then subjected to GO analysis. The 1,445 TSA(–)-specific DEGs could be considered to be genes that were corrected in their expression in TSA(+) embryos to the levels of IVF embryos. The top two GO terms of their gene functions were “regulation of transcription” and “transcription”, which accounted for 13.1% and 10.9% of the listed genes, respectively, with extremely low *P-*values (Supplementary Table S2). The 2,147 TSA(+)-specific DEGs, thought to be inversely affected by TSA treatment, were classified into three GO terms, “chromosome segregation”, “cytokinesis” and “proteolysis involved in cellular protein catabolic process” (Supplementary Table S3).

The question of whether this result is applicable to cloned embryos produced from other types of donor cells remained unanswered. To address this question, we examined the GO terms from TSA(–) and TSA(+)-specific DEGs in 2-cell stage embryos cloned from immature Sertoli cells. GO terms from TSA(–)- and TSA(+)-specific DEGs (1,573 and 1,159 genes, respectively) are shown in Supplementary Tables S4 and S5. The trend for GO terms was slightly different from that of cumulus-cloned embryos, but terms such as “regulation of transcription, DNA-dependent” or “positive regulation of transcription, DNA-dependent” were included in the list. The terms “regulation of transcription, DNA-dependent” accounted 8.8% of the listed genes, which indicated that a number of genes associated with transcriptional regulation were included in this gene list. These results suggest that the genes related to transcriptional regulation improved by TSA treatment in SCNT embryos may also apply to other type of nuclear donor cells.

### Extraction of significant zygotically active genes corrected by TSA treatment

Because the GO analysis described above indicated that TSA treatment effectively activated genes annotated to “regulation of transcription” or “transcription” (Supplementary Table S2), we expected that TSA treatment might facilitate *de novo* RNA transcription during ZGA. To refine the gene list, we extracted the putatively significant zygotic genes using the following conditions: 1) ZAGs expressed higher in the IVF than in the OC (8,067 genes in Fig. 2a); 2) transcripts equivalently expressed in the TSA(+) and IVF but repressed in the TSA(–) (853 genes in Fig. 2b); and 3) factors involved in “regulation of transcription” or “transcription” in GO terms. Finally, 43 genes were extracted by applying these conditions (Supplementary Table S6). These might be the key factors for the improved development of SCNT embryos. To test this possibility, we selected one gene from the list and used it for further experimental analysis. We hypothesized that the candidates might be transcription factors (TFs) because of their multifaceted roles, and that they would be expressed at early stages of development to exert their broader effects as upstream genes. Based on these criteria, we selected a single candidate gene, *Spic* (Spi-C TF, NM_011461), which encodes a TF belonging to the erythroblast transformation-specific (ETS) domain containing TFs and is expressed early during ZGA^3^.

**Figure 3:**
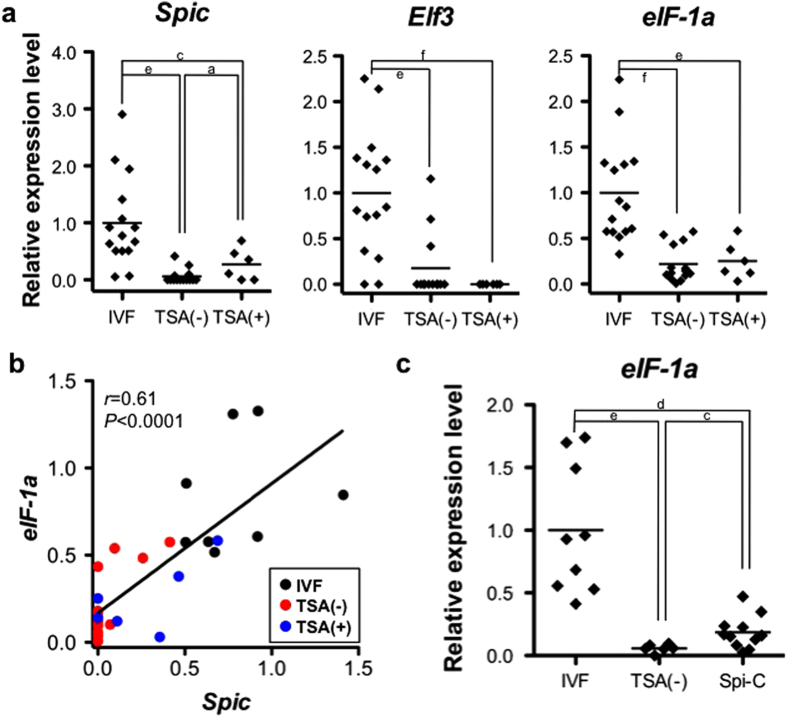
Gene expression levels of ETS-TF family genes and the *Spic* downstream gene, *eIF-1a*. (**a**) Quantitative analysis by quantitative RT–PCR of *Spic, Elf3* and *eIF-1a* expression levels in single 2-cell embryos. The mean expression level for IVF-derived embryos was set at 1.0. The expression level of *Spic* was slightly improved in SCNT embryos following TSA treatment. Values in the same graph with different letters are significantly different (unequal variance Welch’s *t* test). (**b**) Correlation of expression levels between *Spic* and *eIF-1a* in 2-cell embryos. There was a positive correlation between the expressions of both genes (*r* = 0.61; *P* < 0.0001). (**c**) Expression levels of *eIF-1a* in IVF, TSA(–) and Spi-C mRNA-injected 2-cell cloned embryos (Spi-C NT). The mean expression level of IVF-derived embryos was set at 1.0. Values with different letters are significantly different. The injection of Spi-C mRNA into SCNT embryos induced upregulation of *eIF-1a* expression, suggesting that the key TFs induced by TSA treatment could activate downstream genes in SCNT embryos. a, b, c, d, e and f indicate *P* < 0.05, <0.01, <0.005, <0.001, <0.0005 and <0.0001, respectively in Fig. **a** and **c**.

We then did further experimental analysis to test whether *Spic* was one of the key genes responsible for the TSA-dependent effects in SCNT embryos. First, we measured the expression levels of *Spic* in 2-cell SCNT embryos with or without TSA treatment by quantitative RT–PCR. As expected, the TSA(+) embryos showed a significantly improved expression level of *Spic*, although it was still lower than that of IVF-derived embryos (Fig. 3a). By contrast, the expression level of *Elf3*, which belongs to the same ETS-TF gene family as *Spic*^4^, remained at the basal level (Fig. 3a), consistent with our finding that *Elf3* was not a TSA-responsive gene (not in the list in Supplementary Table S6). We also examined the expression level of *eIF-1a*, a translation initiation factor gene known to be one of the downstream genes of *Spic*^5^. Although TSA treatment did not increase the expression level of *eIF-1a* to a statistically significant level, the one-to-one plotting of the *Spic* and *eIF-1a* levels in each embryo revealed a positive correlation between the two (*r* = 0.61, *P* < 0.0001; Fig. 3b). This was a clear indication that TSA improved the expression of TFs, which then lead to activation of their downstream genes. To test this assumption, we injected Spi-C mRNA into TSA(–) SCNT embryos and examined the expression levels of *Spic* and *eIF-1a*. As expected, the expression level of *eIF-1a* increased in SCNT embryos when Spi-C mRNA was injected, while its level was still lower than that of IVF-derived embryos (Fig. 3c). To examine the distribution of the products of Spi-C mRNA, we injected Spi-C–green fluorescent protein (GFP) chimeric mRNA into IVF-derived zygotes and observed GFP expression using fluorescence microscopy. Chimeric Spi-C–GFP proteins were translated 2 h after introduction into the cytoplasts and their signals were localized in the pronuclei (PN), indicating their typical distribution as TFs. At 5 h after injection, the signal was further strengthened in the pronuclei (Supplementary Fig. S2) and could be observed consistently until the 2-cell stage. Thus, it is very probable that the injected Spi-C mRNA was translated shortly after injection and that Spi-C proteins were localized in the PN correctly. Taken together, we postulate that *Spic* potentially plays a key role in the normalization of gene expression cascades in SCNT embryos.

### Improvement of embryo development following the introduction of Spi-C mRNA

**Figure 4:**
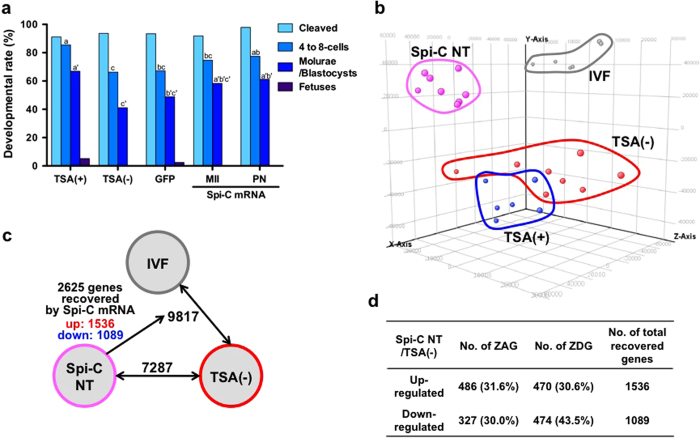
Effects of Spi-C mRNA introduction in the development and gene expression profiles of SCNT embryos. (**a**) Comparison of developmental efficiency of SCNT embryos after the introduction of synthesized mRNA. Different letters indicate statistically significant (*P* < 0.05, Fisher’s exact test). Spi-C mRNA introduction in PN stage improved development into 4- to 8-cell embryos and morula/blastocyst stages. (**b**) PCA of IVF, TSA(–), TSA(+) and Spi-C NT embryos. Spi-C NT embryos were clearly separated from TSA(–) and TSA(+) SCNT embryos. (**c**) Relationships of DEGs with 2-cell stage IVF, TSA(–) and Spi-C NT embryos. Statistical analysis was reperformed with only 2-cell stage embryos (IVF, TSA(–), TSA(+) and Spi-C NT) using the Tukey HSD *post hoc* test (*P* < 0.01). The number of DEGs between TSA(–) and Spi-C NT was 7,287, and their GO terms are shown in Supplementary Table S8. 9,817 genes were differentially expressed between IVF and TSA(–), and 2,625 (1,536 up- and 1,089 down-regulated in Spi-C NT) genes were recovered by Spi-C mRNA injection. (**d**) Numbers of ZAGs and ZDGs that were recovered from TSA(–) embryos by the injection of Spi-C mRNA. The treatment with Spi-C mRNA improved the gene expression levels of aberrant ZAGs and ZDGs in TSA(–) SCNT embryos.

We then examined whether injection of Spi-C mRNA into SCNT embryos would improve their developments *in vitro* and *in vivo*. In a preliminary study, we found that 10 μg/μl Spi-C mRNA injected at the meiosis (M) II or PN stage did not affect the development of fertilized embryos generated by intracytoplasmic sperm injection (ICSI). All embryos were cleaved the next day and 14/15 (93%) of them developed into the morula/blastocyst stage by 72 h in culture (Supplementary Table S7). Then, Spi-C mRNA was injected into SCNT embryos at different concentrations ( < 100 μg/μl) and at different stages (MII or PN). When Spi-C mRNA was injected into SCNT embryos at concentrations of 100 or 10 μg/μl, many embryos arrested at the 2-cell stage and very few developed into morulae or blastocysts. The best results were obtained when 0.1 μg/μl mRNA was injected at the PN stage with significantly higher 4- to 8-cell rates (lower 2-cell arrest rate) than noninjected SCNT embryos (Fig. 4a, Supplementary Table S7). The morula/blastocyst development rate was also higher than in noninjected SCNT embryos (Fig. 4a, Supplementary Table S7). These results suggest that the introduction of Spi-C mRNA into SCNT embryos partially rescued their developmental arrest at the 2-cell stage probably by facilitating ZGA, leading to better development *in vitro*.

We examined the *in vivo* development of SCNT embryos injected with Spi-C mRNA (Spi-C NT embryos) by transferring them into recipient pseudopregnant females. Very few of the embryos developed to term without TSA, irrespective of the mRNA injection dose used (Fig. 4a; Supplementary Table S7).

### Improvement in zygotically activated and degraded gene expression in SCNT embryos by Spi-C mRNA introduction

To explore the possible reasons for improvement of the developmental efficiency of SCNT embryos injected with Spi-C mRNA (Spi-C NT embryos), we carried out global gene expression analysis using Spi-C NT 2-cell embryos. In cluster analysis, Spi-C NT embryos were classified into the SCNT group, but were clearly separated from TSA(–) and TSA(+) embryos (Fig. 1a). This classification was supported by PCA using 2-cell embryos (IVF, TSA(–), TSA(+) and Spi-C NT) (Fig. 4b). Statistical analysis of the 2-cell embryo groups using ANOVA and a statistical post hoc test at *P* < 0.01 showed that 7,287 genes were differentially expressed between TSA(–) and Spi-C NT embryos (Fig. 4c). This gene list included GO terms associated with cell division (“cell cycle” or “mitotic cell cycle”) or RNA processing (“mRNA processing” and “mRNA metabolic process”) (Supplementary Table S8). To understand in more detail the gene functions changed by Spi-C mRNA injection, we next compared the aberrant genes in Spi-C NT with those of TSA(–): 2,625 out of 9,817 DEGs between TSA(–) and IVF showed improved expression levels in Spi-C NT embryos. These recovered DEGs were classified into 1,536 upregulated and 1,089 downregulated genes (Fig. 4c) and each showed characteristic enrichment for GO terms (Supplementary Tables S9 and S10). The upregulated DEGs had a significant association with “positive regulation of transcription” and “positive regulation of RNA metabolic processes”, while the downregulated DEGs were more strongly associated with “cellular macromolecule catabolic process”. Notably, more than half of these DEGs recovered in Spi-C NT embryos were ZAGs or ZDGs, which were defined in the microarray experiments above (see Supplementary Fig. S1, Fig. 4d). We also confirmed by quantitative RT–PCR that Spi-C mRNA injection resulted in a significant upregulation of *eIF-1a*, a translation-initiation factor that plays critical roles in early embryos (see above) (Fig. 3c). These findings supported our notion that *Spic* was one of the upstream key genes that could govern the MZT gene activation cascades.

**Figure 5:**
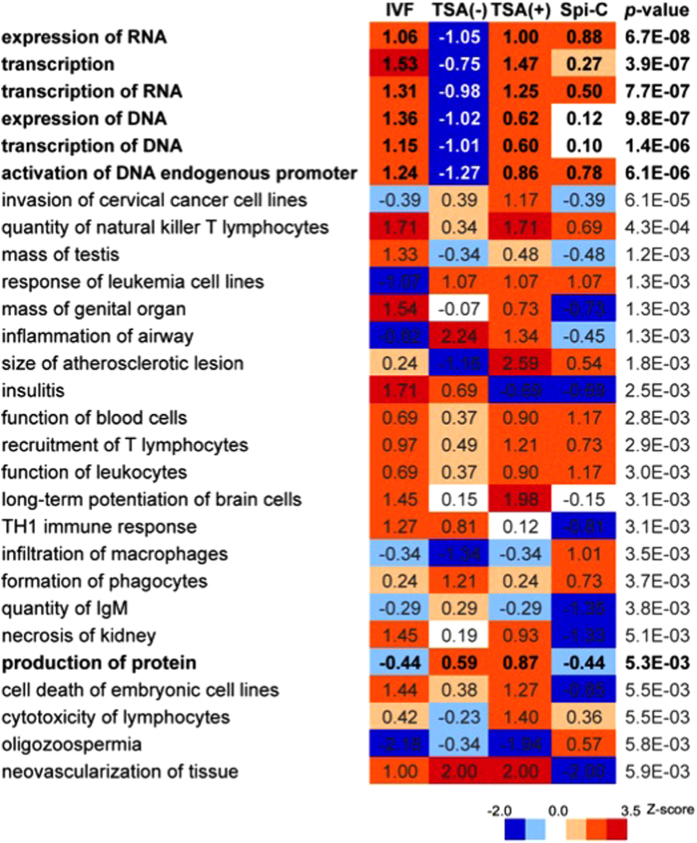
Bioinformatic functional analyses of genes activated in 2-cell IVF-derived embryos showing *z*-score comparisons between IVF, TSA(–), TSA(+) and Spi-C NT embryos. Biological functions enriched in the top 500 ZAGs are arrayed according to *P*-values (*P* < 0.005). *Z*-scores are coloured according to the key in the figure. Functions that were expected to be responsible for the development of early stage embryos (presented with bold letters) were repressed in TSA(–) SCNT embryos, but were almost completely or partially improved by TSA treatment or by Spi-C mRNA injection.

Thus, it is noteworthy that introduction of a single gene’s mRNA significantly improved the broad range of downstream genes in SCNT embryos, but unfortunately its effect was not strong enough to improve embryo development to term. To evaluate this deficiency, we performed Ingenuity Pathway Analysis (IPA) for the list of top 500 ZAGs and calculated *P*-values and *z*-scores of biological functions from normalized expression values of IVF, TSA(–), TSA(+) and Spi-C NT embryos. Figure 5 lists the biological functions enriched significantly in 500 ZAGs (*P* < 0.005) and indicates that the impaired functions in SCNT embryos were largely restored by TSA treatment, and partially improved by Spi-C mRNA injection. In particular, treatment with Spi-C mRNA positively activated functions expected to be responsible for early embryonic gene activation such as “expression of RNA” (–1.05 vs. 0.88 in TSA(–) or Spi-C NT embryos, respectively), “transcription” (–0.75 vs. 0.27), “transcription of RNA” (–0.98 vs. 0.5) and “expression of DNA” (–1.02 vs. 0.12), although these values were smaller than those of TSA-treated SCNT embryos. In addition, network analysis showed that these functions were supported by a few dozen molecules activated by TSA treatment (Supplementary Fig. S3). Thus, our functional analysis suggested that upregulation of *Spic* alone was not sufficient to substitute for the effects of TSA, whereas it may be one of the potent TFs accounting for the improvement of *de novo* RNA synthesis in TSA-treated SCNT embryos.

## Discussion

The treatment of SCNT embryos with HDAC inhibitors represents one of the greatest breakthroughs in cloning research. Although a number of previous studies provided evidence that treatment with HDAC inhibitors improves gene expression activity, their results were predominantly based on immunocytochemistry, so information on the genetic mechanisms involved was limited^8^. Here, we carried out microarray analysis of the gene expression profiles in TSA-treated SCNT embryos to evaluate how TSA improved their developmental ability. Unexpectedly, analysis at the global gene expression level (clustering analysis, and PCA) and the numbers of DEGs (vs. IVF embryos) failed to discriminate TSA-treated SCNT embryos from nontreated SCNT embryos. Nevertheless, we identified a relatively small set of genes (853 downregulated and 592 upregulated) with expression levels specifically improved by TSA treatment. GO analysis of these genes indicated that they were predominantly classified into sets associated with transcription and the regulation of transcription, which might explain the significant improvements in the development of SCNT embryos by TSA treatment. Consistent with this, a previous transcriptome analysis of mouse 2-cell SCNT embryos revealed that TFs and transcriptional regulators were the largest categories of the impaired genes^6^. Furthermore, a recent single-embryo RNA sequencing analysis demonstrated that the early development stages in mice were categorized into some major modules and one of the most important pathways in terms of ZGA was the regulation of transcription^9^. We also demonstrated that TSA treatment strongly activated genes responsible for transcription in SCNT embryos. Recently, Bui *et al.* also indicated that HDAC inhibitor treatment activated ribosomal DNA transcription in late 2-cell stage SCNT embryos up to the level of fertilized embryos^6^. Taken together, it is reasonable to assume that TSA treatment specifically corrected the expressions of a relatively small subset of genes related to transcription, leading to improvements in ZGA and subsequent enhancement of the developmental ability of SCNT embryos. One research group has very recently reported that reprogramming resistant regions (RRRs) identified by SCNT experiments were associated with a repressive histone mark, histone H3 lysine 9 trimethylation (H3K9me3)^7^. Genes within RRRs were reactivated in SCNT embryos following injection with the *Kdm4d* mRNA for H3K9me3 demethylase. Interestingly, the genes responsive to *Kdm4d* included *Spic* and other genes for TFs that were improved by TSA treatment of SCNT embryos in the present study. Therefore, it is very likely that TSA-induced hyperacetylation promotes removal of the repressive epigenetic marks of these histones, leading to improvement of the expression patterns of transcription-related genes. Indeed, according to their H3K9me3-depletion experiments combined with TSA treatment of Matoba *et al.*^7^, TSA had no synergistic effects on clone development, indicating redundancy between them.

Unexpectedly, a relatively large number of genes (2,147 genes) were downregulated by TSA treatment as compared with IVF embryos (Fig. 2b), and some of their GO terms were associated with cell division (“chromosome segregation” or “cytokinesis”). This might have been caused by the potential cytotoxic effect of TSA, which has long been used as an anti-cancer drug. The first two studies of the improvement of SCNT by TSA identified negative effects of this drug when used at high concentrations or for a long period of time^1^. TSA treatment can decrease cell proliferation or apoptosis caused by cell arrest in breast cancer cell lines in a dose- and time-dependent manner^8^. Further optimization of the protocol for TSA treatment in SCNT experiments could be possible by analysing the expression of the subset of genes prone to downregulation by TSA.

It is intriguing that the expressions of such a small number of genes with critical roles in embryonic development were improved specifically by TSA treatment. TSA is a potent HDAC inhibitor with a broad spectrum of action, targeting Class I and II HDACs. Therefore, we initially expected that our microarray analysis would extract a large number of genes that were altered in their expression by TSA treatment. According to studies on the anti-tumour activity of TSA, only a relatively small proportion of genes were up- or downregulated in cancer cell lines following treatment with this HDAC inhibitor^0^. The major genes with altered expression levels were involved predominantly in cell cycle/apoptosis and DNA synthesis, although there were slight differences with the cell lines analysed. Thus, TSA may exert differential effects on gene expression according to the type of cells treated. Therefore, it is more plausible to suppose that TSA stochastically induces open chromatin structure in a genome-wide manner and that existing TFs can then activate their targeting genes within the open chromatin regions. In early embryos, the first trigger for ZGA should be the binding of maternally derived TFs to ZGA genes. Some of such genes encode TFs that then activate their downstream genes. These maternally derived TFs would have accumulated within the recipient ooplasm used for SCNT. This scenario might explain why only a subset of genes related to transcription was activated selectively in TSA-treated SCNT embryos. Despite the small number, the effects of those genes were sufficient to improve the developmental potential.

The hypothesis described above could be supported by the second part of our experiments using mRNA for Spi-C, one of the zygotically expressed TFs. We selected *Spic* for the following reasons: 1) *Spic* is one of the upstream factors for *eIF-1a*^2^, a translation initiation factor, which is transcribed at the earliest stage after fertilization^1^ and is severely downregulated in SCNT embryos irrespective of the donor cell type^3^; 2) Spi-C is one of the ETS-TFs that work as TATA-less promoter-driven TFs in early stage mouse embryos. In early stage embryos, TATA-less promoters act for zygotic gene transcription and ETS-TFs are also working on these biological processes^4^; 3) developmental retardation was initiated by Spi-C RNA interference knockdown in zygotes^2^; and 4) our preliminary cloning experiments showed that SCNT embryos derived from a hybrid F1 mouse strain (C57BL/6 × 129/Sv-*ter*) with high developmental efficiency restored the expression level of *Spic* better than in BDF1 strain SCNT embryos (Supplementary Fig. S4). As expected, SCNT embryos injected with Spi-C mRNA showed an enhanced expression of *eIF-1a* with a positive correlation between the two (Fig. 3b). Furthermore, SCNT embryos injected with Spi-C mRNA showed a better development rate than noninjected SCNT embryos, at least *in vitro*.

Although the details of the function of Spi-C in early stage embryos remains unclear, it has been reported that *Spic* downregulation resulted in reduced expression of genes including *eIF-1a* and *Oct4,* which are expected to play significant roles in preimplantation embryos^2^. Additionally, Spi-C is a regulator of apoptosis resistance^5^ and histone modifications^6^, especially histone H3 lysine 9 dimethylation (H3K9me2), which induces chromatin condensation and gene suppression by removal of histone acetylation from transcriptional regulatory regions. In progenitor B cells, Spi-C expression reduced the H3K9me2 levels within the IgH 3′ regulatory region and promoted their differentiation *in vitro*^6^. These cell metabolic or epigenetic regulations by Spi-C might have contributed to the improved preimplantation development of embryos injected with Spi-C mRNA.

Unfortunately, treatment with Spi-C alone was not sufficient to improve the *in vivo* development of SCNT embryos. As the genes that were improved in their expression by TSA treatment included many transcriptional factors other than Spi-C (Supplementary Table S6), some of them could regulate ZGA by other pathways. Induced pluripotent stem cells were established successfully by the introduction of three to four TFs activated in embryonic stem cells, into differentiated somatic cells^7^. Similarly, it is possible that the development of SCNT embryos would be improved by the introduction of two or more TFs. *Ifng*, *Hipk1* or *Ppp1r8* (Supplementary Table S6) could be candidates because they are TFs that are highly transcribed specifically in early embryos, are reactivated by injection of mRNA for H3K9me3 demethylase *Kdm4d*^7^ and seem to work independently of *Spic* according to a pathway analysis.

In conclusion, here we succeeded in extracting 1,445 differentially expressed genes by comparing the global gene expression patterns of TSA-treated SCNT embryos with nontreated embryos. Zygotic genes upregulated by TSA treatment were enriched with those encoding for cell components involving transcriptional activity. Although Spi-C mRNA introduction into SCNT embryos could not improve their developmental ability in terms of the birth rates *in vivo*, the developmental efficiency *in vitro* was improved significantly by correction of the expression patterns of genes related to zygotic gene activation and degradation. These data indicate that TFs might be critical targets for future technical improvements in cloning by SCNT in mammals.

## Methods

### Animals

Eight- to 10-week-old (C57BL/6 × DBA/2) F1 (BDF1) female mice (Japan SLC, Inc., Shizuoka, Japan) were used for the collection of the recipient oocytes and as SCNT donors. Eight- to 12-week-old ICR strain pseudopregnant female mice (CLEA Japan, Inc., Tokyo, Japan) were used as embryo transfer recipients. The animals were provided with water and commercial laboratory mouse chow *ad libitum* and housed under controlled lighting conditions (daily light from 07:00–21:00). They were maintained under specific-pathogen-free conditions. All animal experiments were carried out in accordance with the approved guidelines of the RIKEN BioResource Center. All experimental protocols were approved by the Animal Care and Use Committee of RIKEN BioResource Center.

### Nuclear transfer

Nuclear transfer was carried out as described^0^. Briefly, BDF1 female mice were induced to superovulate by the injection of 7.5 IU of pregnant mare serum gonadotropin (Sankyo Yell Yakuhin, Co., Tokyo, Japan) and 7.5 IU of human chorionic gonadotropin (hCG; ASKA Pharmaceutical Co., Ltd, Tokyo, Japan) at a 48 h interval. At 15 h after the hCG injection, cumulus–oocyte complexes were collected from the oviducts and the cumulus cells were dispersed in potassium-enriched simplex optimized medium (KSOM)^8^ containing 0.1% bovine testicular hyaluronidase. Collected cumulus cells were used as nuclear donors. Immature Sertoli cells were collected as described previously and used as nuclear donors^9^. The oocytes were enucleated in Hepes-buffered KSOM containing 7.5 μg/ml cytochalasin B. The donor nuclei were injected into enucleated oocytes using a Piezo-driven micromanipulator (PMM-150FU, Primetech, Ibaraki, Japan). After culture in KSOM for 1 h, the injected oocytes were activated in Ca^2+^-free KSOM containing 2.5 mM SrCl_2_ for 1 h. The reconstructed embryos were cultured in KSOM containing 5 μg/ml cytochalasin B for 5 h. For TSA treatment, 5 nM was added into each medium from activation for 8 h.

### Embryo transfer

Reconstructed embryos that had reached the 2-cell stage after 24 h in culture were transferred into the oviducts of pseudopregnant ICR female mice on day 0.5 (the day following sterile mating with a vasectomized male mouse). On day 19.5, pups were delivered by caesarean section and the live ones were nursed by lactating ICR strain female mice.

### RNA preparation, amplification and hybridization

Labelled RNA samples were prepared as described^0^. Total RNA was extracted with TRIzol (Life Technologies, Carlsbad, CA, USA) from 10 cloned 2-cell embryos cultured for 24 h. RNA was subjected to two rounds of linear amplification using TargetAmp Two-Round Aminoallyl-aRNA Amplification Kits (Epicentre, Madison, WI, USA) according to the manufacturer’s instructions. For oocytes, control 2-cell embryos and nuclear donor cells, 10 pooled MII oocytes, 10 pooled IVF-derived 2-cell embryos and 500 cumulus cells were all subjected to total RNA extraction and two rounds of RNA amplification. Amplified RNA was labelled with Cy3 dye (GE Healthcare, Little Chalfont, Buckinghamshire, UK) and hybridized to a Whole Mouse Genome oligo DNA microarray (4 × 44 K; Agilent Technologies) for 16 h at 65 °C according to the manufacturer’s instructions.

### Microarray analysis

Scanning of microarray slides was performed with a DNA microarray scanner at 5μm resolution (Agilent Technologies). Scanned image files were processed to give signal intensities using Feature Extraction software (Agilent Technologies). To extract differently expressed genes, all raw data were loaded into Gene Spring GX 12.5 software (Agilent Technologies) and normalized by default settings. Probes whose values were less than 10 in all wild-type IVF-derived and SCNT embryos were excluded from further analysis (39,870 probes). Normalized values were subjected for the extraction of differentially expressed genes among each group to ANOVA and Tukey *post hoc* statistical analysis at a 1% significance level using the Benjamini and Hochberg procedure. GO analysis was carried out with DAVID Bioinformatics Resources 6.7 ( http://david.abcc.ncifcrf.gov/)^1^ and the 39,870 probes noted above were used as a background gene set.

### Quantitative RT–PCR

The cDNA from single SCNT or IVF-derived embryos was synthesized with Cell to cDNAII (Ambion, Austin, TX, USA). The ABI Prism 7900HT Sequence Detection System (Applied Biosystems, Foster City, CA, USA) was used to determine the level of cDNA. The *Gapdh* gene in each embryo was used as an endogenous reference (4352339E, Life Technologies). Amplifications were carried out with Quantitect Probe PCR kit or Quantitect SYBR Green PCR kit (Qiagen, Hilden, Germany). The primer sets used for quantification were as follows: *Spic*, 5′–acgctaactactatggaatg–3′ and 5′–tttttatgatctccccagtt–3′; *Elf3*: 5′–caatgaaacggacacatttggt–3′ and 5′–agatgtcatcaatgtcttcatcatca–3′; and *eIF-1a*: 5′–caatgaaacggacacatttggt–3′ and 5′–agatgtcatcaatgtcttcatcatca–3′. Statistical analysis was performed using an unequal-variance Welch’s *t* test.

### mRNA synthesis and injection

mRNA was synthesized as described^1^. Full-length cDNA of *Spic* was amplified from 2-cell embryos and cloned into pcDNA4/Myc-HisA (Life Technologies). mRNAs for Spi-C were synthesized by a T7mMESSAGEmMACHINE Kit (Life Technologies) and dissolved in nuclease-free water. Spi-C mRNA was injected by a Piezo-driven micromanipulator into cytoplasts of oocytes or pronuclear stage embryos.

### Bioinformatic network analysis

To understand more completely the functions of genes restored by TSA treatment or mRNA introduction, bioinformatic network analysis was performed using IPA (Qiagen). The top 500 ZAGs expressed differentially between OC and IVF (Fig. 2a) were chosen and loaded into IPA. *P*-values and *z*-scores were calculated using the Diseases and Functions analysis.

## Author Contributions

K.I. and A.O. designed the research. M.O., S.K. and N.O. prepared manipulated embryos and K.I. analysed expression data. T.N. and T.N. designed the vector constructs and synthesized mRNA. K.A. performed the network analysis with IPA K.I. and A.O. prepared the manuscript.

## Additional Information

**Accession codes:** The microarray data have been deposited in the Gene Expression Omnibus database (GEO; http://www.ncbi.nlm.nih.gov/geo/) and given the series accession number GSE63886.

**How to cite this article**: Inoue, K. *et al.* Trichostatin A specifically improves the aberrant expression of transcription factor genes in embryos produced by somatic cell nuclear transfer. *Sci. Rep.* **5**, 10127; doi: 10.1038/srep10127 (2015).

## Supplementary Material

Supplementary Information

Supplementary Table S1-S10
