# Supplementary material for: Trichostatin A specifically improves the aberrant expression of transcription factor genes in embryos produced by somatic cell nuclear transfer
Source: Sci Rep. 2015 May 14;5:10127. doi: 10.1038/srep10127 (PMC4431350; doi:10.1038/srep10127)

Supplementary Information

Trichostatin A specifically improves the aberrant expression of transcription factor genes in embryos produced by somatic cell nuclear transfer

Kimiko Inoue, Mami Oikawa, Satoshi Kamimura, Narumi Ogonuki, Toshinobu Nakamura, Toru Nakano, Kuniya Abe and Atsuo Ogura.

Figure S1 | Venn diagrams showing the numbers of differentially and commonly expressed genes among IVF, TSA(–) and TSA(+). Red, blue and green circles indicate differentially expressed genes (DEGs) between 2-cell embryos—IVF, TSA(–) and TSA(+)—and other targets, respectively (**a** and **b**, OC; **c** and **d**, Cumulus). Double and single underlines indicate commonly expressed genes between IVF and TSA(–), and IVF and TSA(+) embryos, respectively. Numbers indicate genes upregulated (**a**) or downregulated (**b**) in 2-cell embryos—IVF, TSA(–) and TSA(+)—than in OC and their differentially and commonly expressed genes. Also shown are the numbers of genes upregulated (**c**) or downregulated (**d**) in 2-cell embryos—IVF, TSA(–) and TSA(+)—than in Cumulus, and their differentially and commonly expressed genes. These results indicate that the numbers of DEGs hardly improved in IVF and SCNT embryos irrespective of TSA treatment.

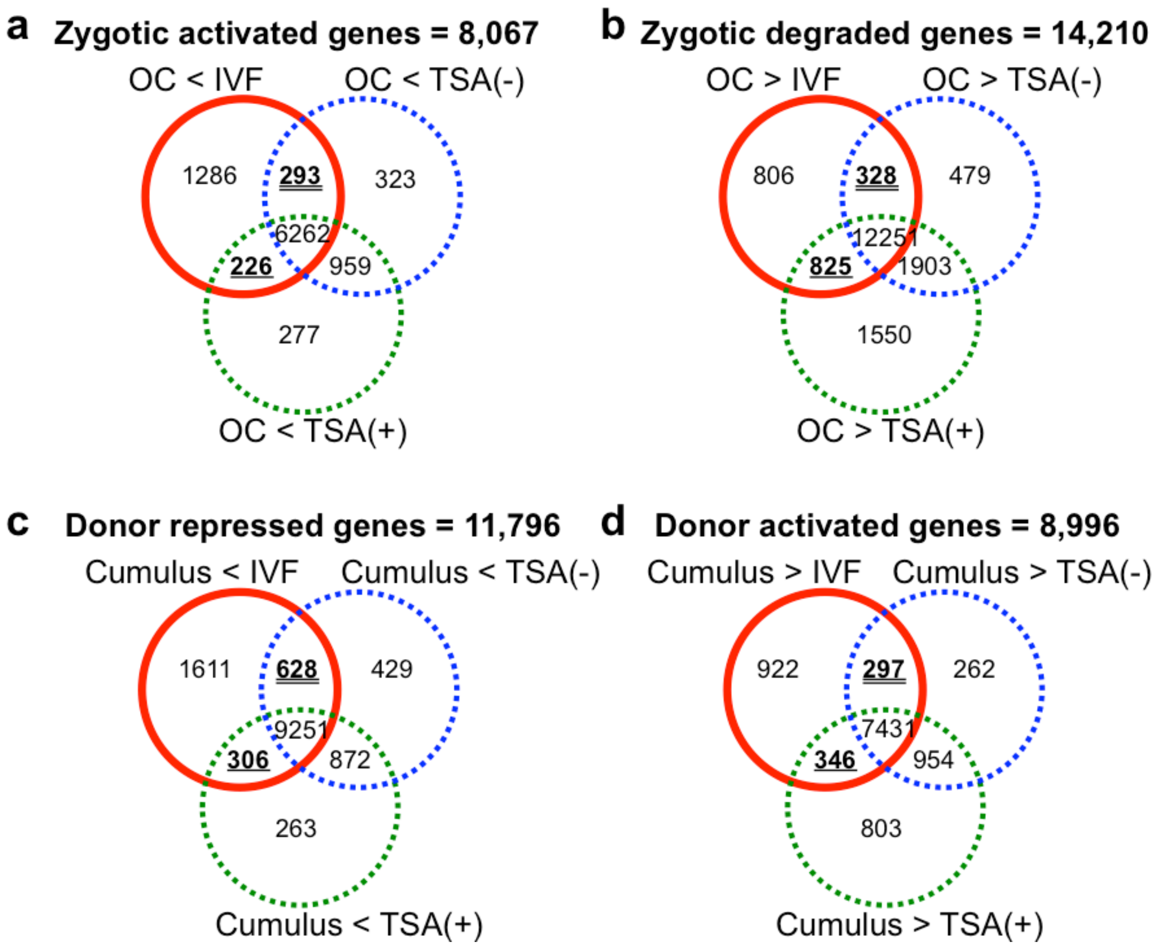

Figure S2 | Pronuclear stage embryos after Spi-C–GFP chimeric mRNA injection. Bright-field image of embryo pronucleus (left), stained by DAPI to show DNA (centre) and Spi-C–GFP localisation (right). Spi-C–GFP mRNA was translated shortly after its introduction into pronuclear stage IVF-derived embryos and its signals were localised to the pronuclei, indicating its typical distribution as a TF.

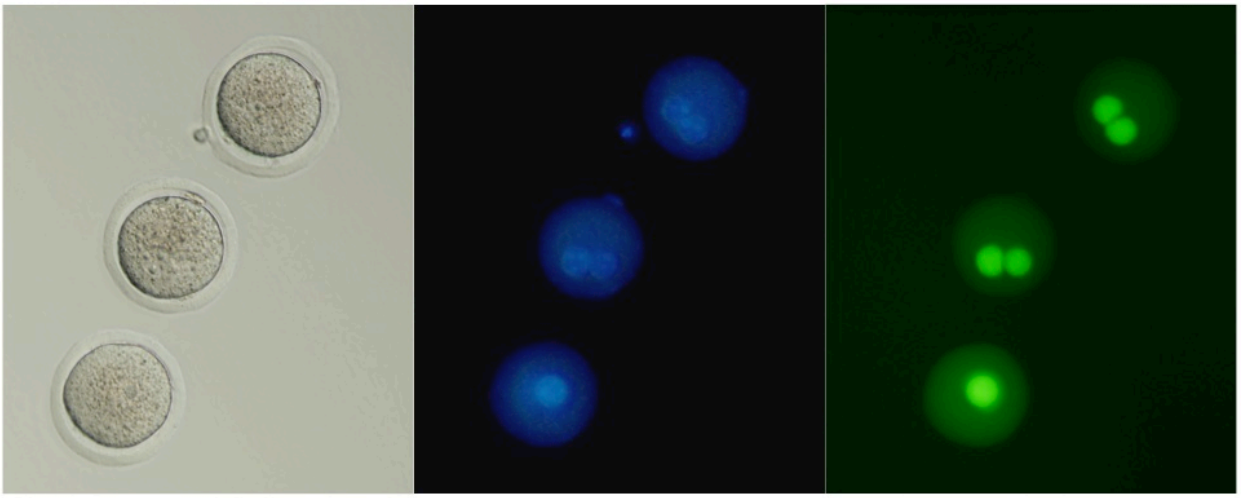

Figure S3 | Network diagrams of "expression of RNA" and "transcription" as biological functions shown in Figure 5. Numbers below shapes indicate the log ratio of expression of genes/molecules. These functions were activated by upregulated molecules in TSA(+) embryos (shown as orange octagons), but they were inhibited in TSA(–) embryos (blue octagons). Spi-C mRNA introduction improved the expression of some of these genes/molecules and partially activated both functions in Spi-C NT embryos. The lower table indicates the number of genes/molecules controlling these biological functions.

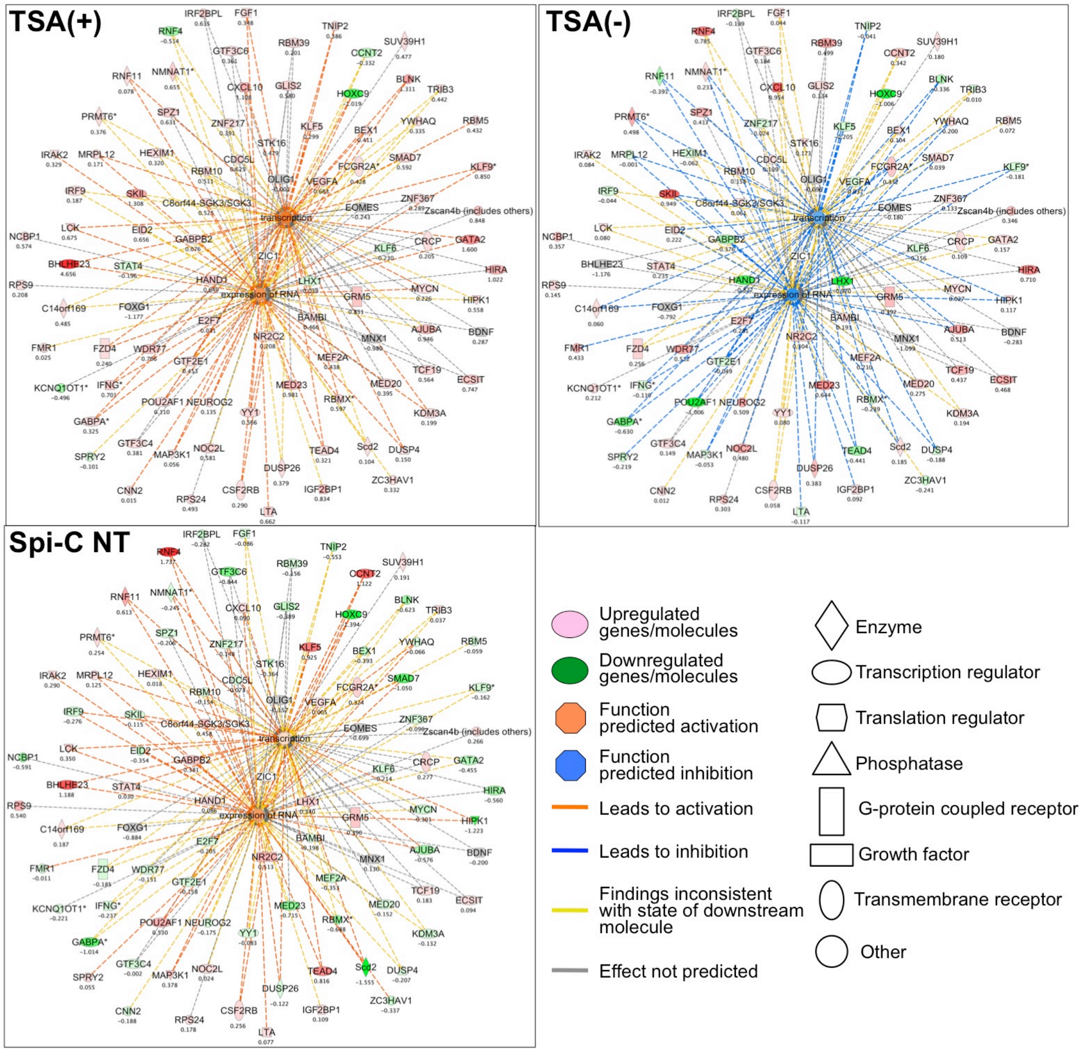

Functions Annotation	Activation z-score	No. of Molecules
expression of RNA	1.055	93
transcription	1.527	84
transcription of RNA	1.306	82
expression of DNA	1.360	71
transcription of DNA	1.152	68
activation of DNA endogenous promoter	1.238	53

Figure S4 | Gene expression levels of *Spic* and *Elf3* in SCNT embryos derived from a hybrid F1 mouse strain (C57BL/6 × 129/Sv-*ter*) (B6 × 129) with high developmental efficiency. The expression level of *Spic* in the B6 × 129 strain embryos was higher than in the BDF1 strain.

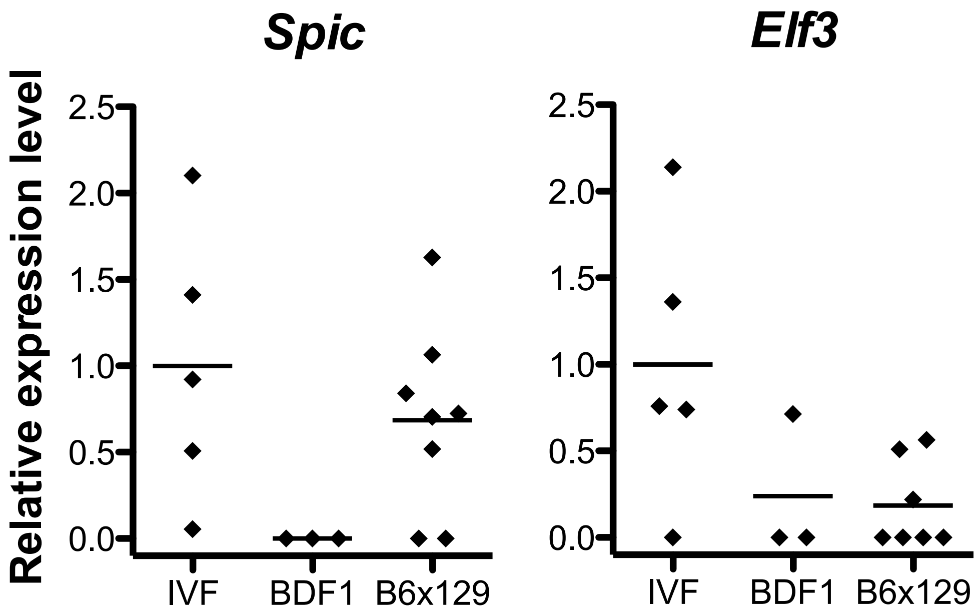

Supplement: Supplementary Information [file srep10127-s1.pdf]
